# Commotio Cordis in Sudden Cardiac Death in the Young: A State-of-the-Art Review

**DOI:** 10.31083/RCM43357

**Published:** 2025-09-18

**Authors:** Cecilia Salzillo, Andrea Marzullo

**Affiliations:** ^1^Department of Experimental Medicine, PhD Course in Public Health, University of Campania “Luigi Vanvitelli”, 80138 Naples, Italy; ^2^Department of Precision and Regenerative Medicine and Ionian Area, Pathology Unit, University of Bari “Aldo Moro”, 70124 Bari, Italy

**Keywords:** commotio cordis, sudden cardiac death, young, autopsy pathology

## Abstract

Commotio cordis is a rare but fatal cause of sudden cardiac death in young people, particularly athletes exposed to non-penetrating chest trauma. Commotio cordis occurs when an impact to the chest triggers a lethal ventricular arrhythmia in the absence of pre-existing structural heart disease. Despite advances in the understanding of commotio cordis, the prevention and management of this condition remain challenging. The literature indicates that commotio cordis is most common in adolescents and sports such as baseball, football, and ice hockey. The key pathogenic mechanism involves a chest impact occurring during a vulnerable phase of the cardiac cycle, leading to ventricular fibrillation. Immediate cardiopulmonary resuscitation and prompt use of an automated external defibrillator are crucial for survival. However, the effectiveness of preventive measures, such as chest protectors and greater awareness of cardiovascular emergencies, remains debated. As a leading cause of sudden death in young athletes, commotio cordis requires further research to refine prevention strategies and improve outcomes. This review provides an updated overview of the pathophysiological mechanisms, risk factors, intervention strategies, and preventive approaches for this condition.

## 1. Introduction 

Commotio cordis (CC) is a rare but dramatic and often fatal cardiovascular phenomenon resulting from direct blunt trauma to the chest wall. It represents a significant cause of sudden cardiac death in the young (SCDY), particularly among athletes participating in contact sports, in the absence of pre-existing structural heart disease [1, 2]. CC arises following a direct, non-penetrating thoracic impact that acts as an arrhythmogenic trigger during a specific vulnerable phase of the cardiac cycle, most notably the ascending phase of the T wave, leading to ventricular fibrillation and immediate haemodynamic collapse [3, 4].

Although traditionally considered a rare entity, the true incidence of CC is likely underestimated, especially within youth sporting environments, where fatal episodes may go unrecognised or be misattributed. Its epidemiological relevance is substantiated by autopsy series and national out-of-hospital cardiac arrest registries, which consistently report a predominance among adolescent males engaged in sports such as baseball, American football, and ice hockey [3, 5, 6, 7, 8], and in a significant percentage it occurs in non-sports contexts, such as assaults, car accidents and daily activities, with a wider age range and a higher percentage of females [2].

From a pathophysiological standpoint, the induction of lethal arrhythmias in CC appears to depend on several key variables: the kinetic energy of the impact, its precise location over the precordium, the timing within the cardiac cycle, and mechanotransduction mechanisms at the myocardial level. These mechanisms are thought to involve the activation of stretch-sensitive ion channels, contributing to electrical instability [9, 10]. Despite the absence of underlying structural cardiac abnormalities, the condition carries a high fatality rate in the absence of immediate cardiopulmonary resuscitation (CPR) and prompt defibrillation.

In this review, we critically examine the current state of knowledge surrounding commotio cordis, with particular focus on its pathophysiology, epidemiology, and medicolegal implications in the context of SCDY. Additionally, we discuss contemporary prevention strategies and offer practical recommendations for the on-field management of such events, informed by the most recent guidelines and available evidence.

## 2. Literature Review 

### 2.1 Epidemiology

CC is regarded as a rare condition but constitutes a significant cause of SCDY, particularly among otherwise healthy individuals engaged in sporting activities. According to data from the US Commotio Cordis Registry, which systematically collects documented cases at the national level in the United States, the incidence is estimated to be approximately 15–25 cases per year. However, this figure is likely an underestimation, due to the inherent challenges in post-mortem identification in the absence of structural cardiac abnormalities [11, 12].

Prevalence varies considerably across countries, reflecting geographical, cultural and systemic differences in case reporting and diagnostic accuracy. In many regions, the absence of dedicated national registries and the lack of standardised autopsy protocols hinder the precise identification and classification of cases. Moreover, the association of CC with specific sports, such as baseball in the United States, ice hockey in Canada, and rugby in Europe, contributes to the geographical heterogeneity observed in the distribution of this pathology [13].

CC exhibits a clear male predominance, with the average age of affected individuals ranging between 12 and 18 years [14]. This distribution is attributed to greater male participation in high-impact thoracic sports, as well as to hormonal and physiological factors that may influence the electrical susceptibility of the myocardium [15].

Contact sports, including baseball, American football, lacrosse, ice hockey, and, increasingly, association football and rugby, are most frequently associated with CC. Nevertheless, isolated cases have also been reported in non-sporting contexts, such as schoolyard incidents or recreational play, highlighting the relevance of this phenomenon even beyond the competitive sporting environment [16].

Furthermore, documented cases suggest that even combat sports such as karate, taekwondo and mixed martial arts may constitute risk contexts for CC, as blows to the chest may accidentally coincide with the vulnerable phase of the cardiac cycle [16]. Although less frequent, such events are clinically relevant, especially considering the growing popularity of these disciplines among adolescents and young adults.

Particular attention must be given to the school-aged and youth athletic population, within which CC represents one of the leading causes of sudden death occurring on the field. The absence of prodromal symptoms and the rapid clinical progression underscore the importance of early identification of at-risk individuals and the implementation of appropriate emergency response protocols [17].

### 2.2 Pathophysiological Mechanisms

**Fig. 1.**
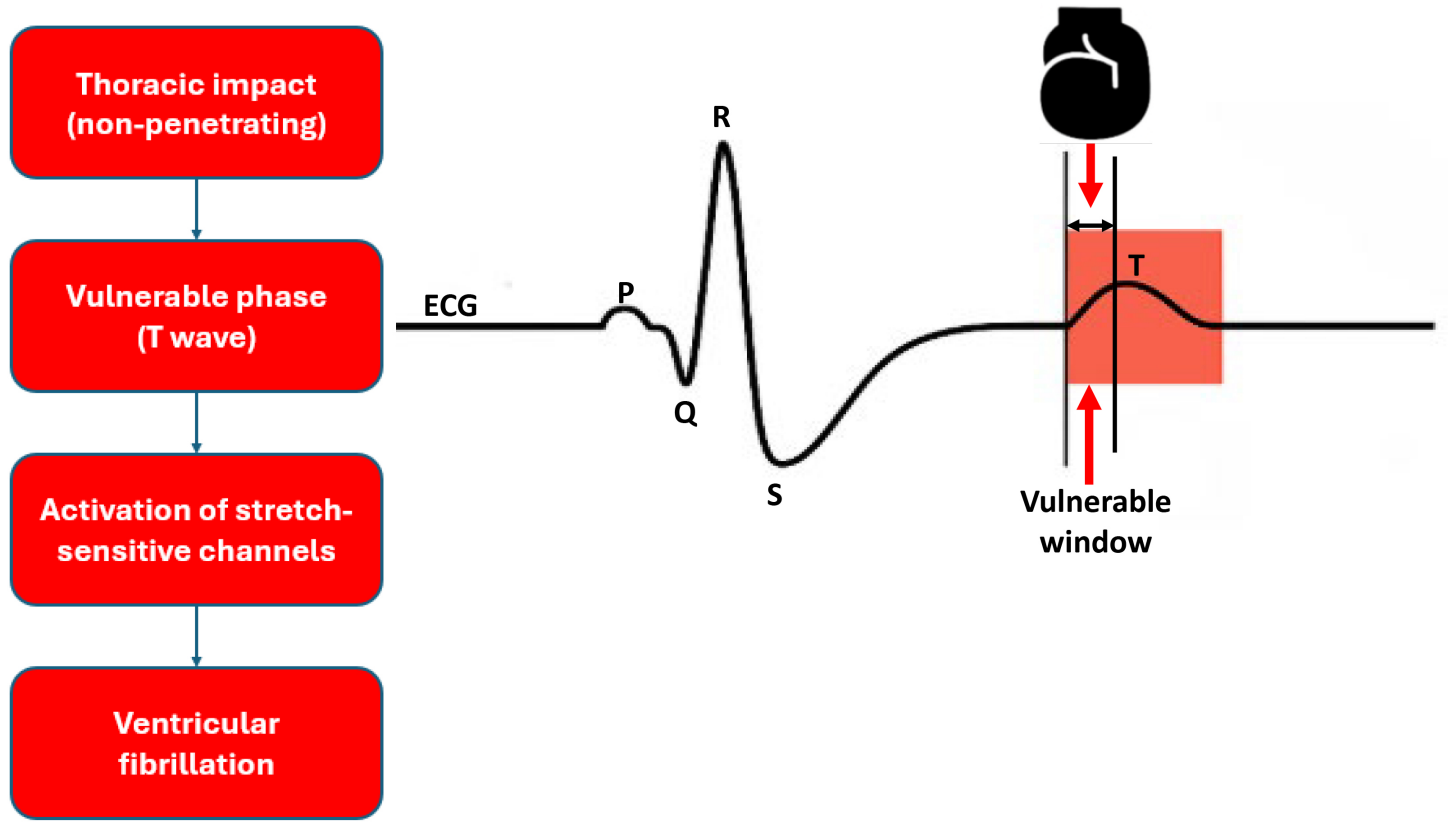
**Pathophysiological mechanism of commotio cordis**. The figure shows a simplified electrocardiogram (ECG) trace. A thoracic impact during the ascending limb of the T wave (vulnerable window) may trigger una fibrillazione ventricolare due to activation of stretch-sensitive ion channels.

CC occurs when a non-penetrating thoracic impact strikes the heart during the ascending phase of the T wave in the cardiac cycle, corresponding to the early phase of ventricular repolarisation. During this period, the myocardium is particularly vulnerable to electrical disturbances, and mechanical trauma can induce ventricular fibrillation (VF) even in the absence of structural cardiac abnormalities (Fig. 1) [18].

Recent investigations have confirmed that the likelihood of VF is significantly increased when the impact occurs within a critical time window of approximately 10–20 milliseconds during repolarisation [19]. Experimental studies, alongside computational simulations, have further clarified the temporal dynamics of electrophysiological events that contribute to myocardial electrical destabilisation following mechanical stimulation [20, 21, 22].

The traumatic event results in an acute deformation of the myocardial tissue, initiating a process of mechanotransduction (transduction of a mechanical stimulus into an electrical signal) that is, the conversion of a physical stimulus into an electrical signal. This process leads to the activation of stretch-sensitive ion channels (channels that activate in response to mechanical forces on the cell membrane), including adenosine triphosphate-sensitive potassium (K-ATP) channels and members of the transient receptor potential (TRP) channel family, which disrupt the transmembrane potential and facilitate the emergence of abnormal electrical activity [23].

These channels have been shown to induce afterdepolarisations and chaotic propagation of electrical impulses, thereby increasing the myocardium’s susceptibility to VF. Recent studies indicate that the activation of these channels is modulated by both the magnitude of the mechanical stretch and the pre-existing electrophysiological state of the myocardial cells [24, 25].

A defining feature of CC is the absence of morphological abnormalities detectable at autopsy, rendering the diagnosis primarily clinical-anamnestic and one of exclusion. The principal differential diagnoses include hereditary cardiomyopathies, channelopathies, and congenital coronary anomalies [26, 27, 28].

Recent literature highlights the necessity of a comprehensive autopsy, performed using standardised protocols, which should include histopathological examination, toxicological screening, and, where appropriate, molecular genetic testing, in order to exclude alternative causes and substantiate a post-mortem diagnosis of CC [27, 29].

#### Non-immediate Arrhythmias From Blunt Chest Trauma

CC represents the most acute and immediate manifestation of arrhythmias induced by blunt thoracic trauma. The literature also describes cases of ventricular arrhythmias and delayed-onset atrioventricular conduction disturbances. These arrhythmias do not necessarily occur at the time of impact but may emerge in the subacute period or days later, as a consequence of myocardial contusions, tissue microlesions, and local inflammatory processes. These alterations can induce electrical remodeling and ion channel dysfunction, favoring the development of arrhythmias in the absence of obvious structural abnormalities [24, 25].

Unlike ventricular fibrillation typical of CC, which is triggered by a direct impact during a critical time window of the cardiac cycle [3, 18, 19], late-onset arrhythmias from blunt trauma may result from mechanisms of progressive myocardial damage, often underestimated in the initial phase. Persistent activation of stretch-sensitive ion channels, such as K-ATP and TRP channels, already implicated in the genesis of CC [23, 24], may also contribute to the genesis of arrhythmias with a delayed onset. In such cases, prolonged electrocardiographic surveillance, combined with cardiac imaging and genetic testing [26, 27, 28, 29], may be indicated even in the absence of overt symptoms.

Therefore, it is appropriate to extend clinical and diagnostic attention to subacute post-traumatic arrhythmias, especially in young subjects involved in thoracic contusion events, whether athletic or non-athletic.

### 2.3 Postmortem Diagnosis and Medico-Legal Implications 

Post-mortem diagnosis of CC is inherently challenging, as the event typically occurs in the absence of structural abnormalities detectable at autopsy. The only suggestive feature may be a history of recent blunt thoracic trauma, temporally associated with sudden death in a young, previously healthy individual [30, 31, 32].

It is essential to conduct a rigorous differential diagnosis with other causes of SCDY, such as cardiac channelopathies, which often lack macroscopic pathological findings. Within the spectrum of SCDY, CC constitutes a rare but clinically relevant cause, particularly among male individuals engaged in contact sports. According to data from the National Registry of Sudden Death in Athletes, CC accounts for up to 20% of sudden deaths associated with physical activity during adolescence [6]. Only a comprehensive autopsy, including detailed histopathological analysis and, where appropriate, post-mortem genetic testing, enables the exclusion of alternative diagnoses and supports the identification of probable CC (Table 1).

**Table 1.** **Postmortem differential diagnosis in cases of sudden juvenile death**.

Heart disease	Structural anomalies	Genetic testing	Activity context
Commotio cordis	No	Yes (to exclude alternative diagnoses)	Frequently during sports
Cardiomyopathies	Yes	Yes	During sports activity or exertion
Channelopathies	No	Yes	During sports activity or exertion
Coronary artery anomalies	Sometimes	No	During sports activity or exertion

Note: The table is for comparison and simplification purposes only. Some conditions may occur in sports settings without being directly caused by them. The presence or absence of structural abnormalities may vary depending on the level of postmortem diagnostic investigation.

From a medico-legal perspective, CC assumes particular significance in the insurance, sporting, and in some cases criminal domains [33]. The identification of the event and its temporal and contextual correlation with a regulated sporting activity may have legal implications, including civil or professional liability [34].

In many countries, current regulations mandate the presence of life-saving equipment in sporting venues, including automated external defibrillators (AEDs) and personnel trained in CPR. Failure to provide such emergency infrastructure may constitute negligence on the part of managing authorities or coaching staff, especially in educational or competitive settings. Additionally, the presence or absence of chest protectors conforming to approved safety standards may influence legal assessments of liability [2].

Examples described in the literature demonstrate that CC events can lead to significant legal consequences. In one case, a death that occurred during a fight was classified as manslaughter, with CC identified as the cause of death [33]. In another case, the absence of a defibrillator in a sports facility raised questions of civil liability [34].

### 2.4 Prevention

Prevention of CC represents a significant challenge in the management of SCDY among athletes, due to the unpredictable nature of the event and the absence of pre-existing cardiac pathology. According to the guidelines issued by the American Heart Association (AHA) and recommendations from international sporting bodies, the most effective preventive measure is the immediate availability of an AED in all settings where sports activities are conducted. This must be accompanied by mandatory training in CPR for coaches, referees, and any school or medical personnel present on-site. Evidence indicates that defibrillation within three minutes of the traumatic event can increase survival rates to over 70% [35, 36].

In recent years, increased attention has also been directed towards the design and use of chest protectors [37]. In this context, the National Operating Committee on Standards for Athletic Equipment (NOCSAE) [38] has introduced the ND200 standard, which sets minimum performance criteria for equipment capable of attenuating impact forces and reducing the risk of VF. Certain federations, such as US Lacrosse, have mandated the use of such protective devices for high-risk positions (e.g., goalkeepers). However, a study has highlighted that not all commercially available protectors offer sufficient protection against CC, emphasising the need for further biomechanical testing and regulatory revisions to ensure effectiveness (Table 2) [39].

**Table 2.** **Types of chest protection devices for the prevention of commotio cordis**.

Model	Description	Regulatory compliance	Estimated preventive efficacy	Usage context
A*	Standard non-certified protection, made of light material (example, basic foam)	None	Low	Recreational, school-level use
B**	ND200-certified device (NOCSAE), with impact-reduction technology	ND200 (NOCSAE)	Moderate–High	Organised sports (example, lacrosse, football)
C***	Experimental model with multilayered shock absorption and integrated sensors	Under validation	High (in preclinical models)	Pilot studies, biomechanical research

*A: standard non-certified protection. **B: ND200 standard compliant device. ***C: experimental model with multilayer shock absorption. NOCSAE, National Operating Committee on Standards for Athletic Equipment.

Current guidelines also advocate for comprehensive education programmes aimed at athletes and their families, focusing on the early recognition of cardiac arrest and the importance of immediate first aid. The implementation of standardised Emergency Action Plans (EAPs) in schools and sports clubs is regarded as a key best practice to enhance emergency preparedness and reduce mortality associated with commotio cordis [40, 41, 42, 43, 44].

**Fig. 2.**
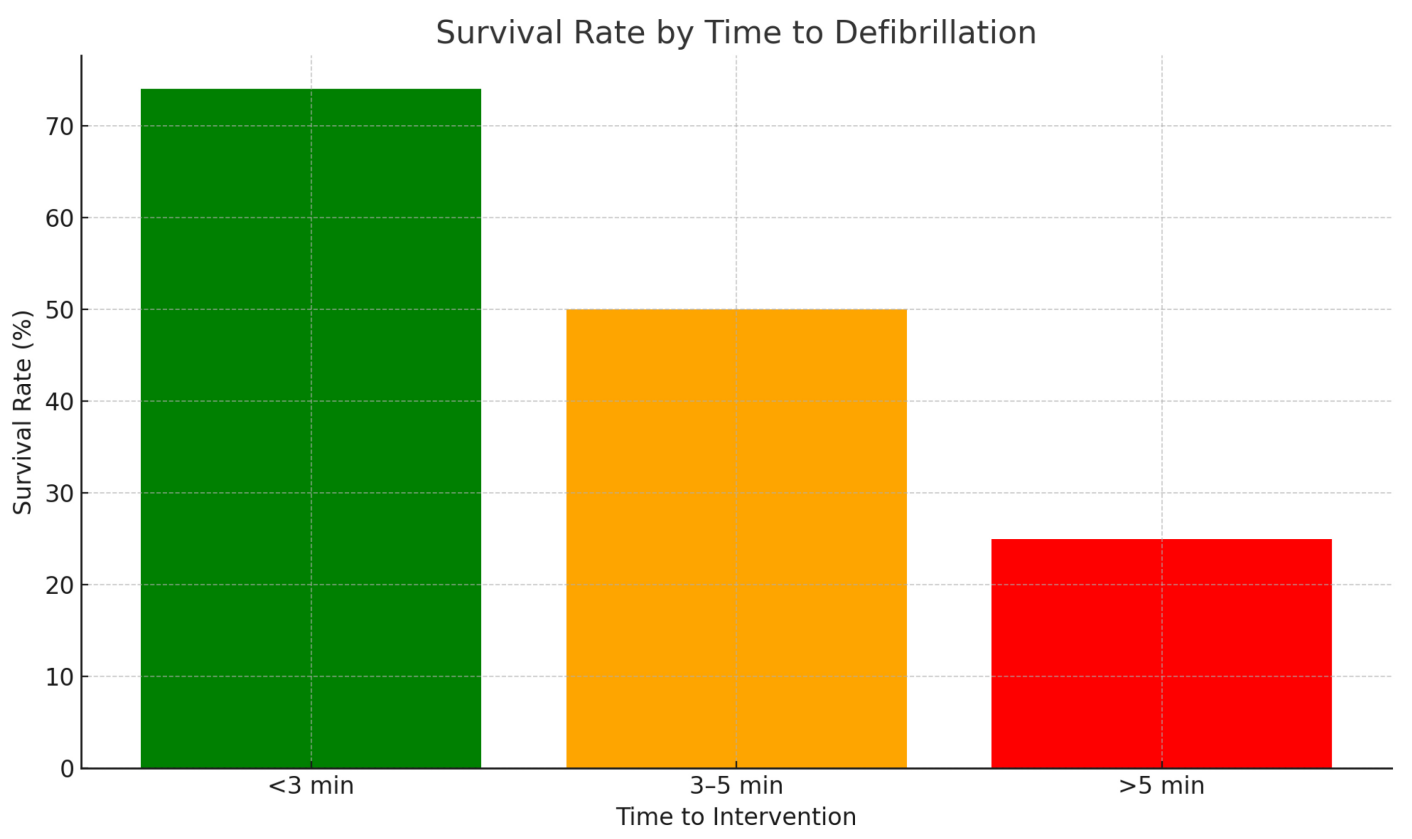
**Survival rates for commotio cordis in relation to timeliness of intervention**. Data adapted from Maron *et al*. [12], Heart Rhythm, 2013.

Fig. 2 (Ref. [12]) describes survival rates from commotio cordis in relation to the timeliness of intervention.

### 2.5 Non-Sporting Contexts

Although CC is traditionally associated with athletic activities, a significant proportion of cases occur in non-sporting settings [2].

A systematic review identified that 36% of 334 documented CC cases transpired outside of sports, with the majority resulting from assaults (76%), followed by motor vehicle accidents (7%) and routine daily activities (16%). In non-sport-related incidents, the causative impacts were predominantly non-projectile, often involving bodily contact (79%), contrasting with the projectile-related mechanisms common in sports settings. Notably, the proportion of female victims was higher in non-sport contexts (13%) compared to sport-related cases (2%). Mortality rates in non-sport CC incidents were significantly elevated (88%) relative to sport-related cases (66%), a disparity attributed to lower rates of bystander CPR (27% vs. 97%) and defibrillation (17% vs. 81%), as well as delayed initiation of resuscitative efforts, with 80% of non-sport cases experiencing delays exceeding three minutes [32].

These findings underscore the necessity for heightened awareness and preparedness for CC in non-athletic environments. Implementing widespread CPR training and ensuring the AEDs in public and domestic settings are critical measures to improve survival outcomes in non-sport-related CC events [45, 46].

### 2.6 Future Directions of Research

CC is a major cause of SCDY in athletes and represents a major challenge for prevention and management. Current knowledge highlights the need to investigate several areas to improve the understanding and prevention of this lethal event.

Development and evaluation of protective devices: future research should focus on optimizing thoracic protective devices. A recent study has shown that many commercially available chest protectors do not provide adequate protection against CC, highlighting the importance of developing more effective standards and evaluating the effectiveness of existing devices through advanced experimental models [47].

Biomechanical modeling and simulations: the use of advanced biomechanical models, such as the Total Human Model for Safety (THUMS) [21], can provide detailed information about the effects of thoracic impacts and help identify conditions that lead to CC. These models can be used to simulate various scenarios and evaluate the effectiveness of different preventive strategies [38, 47, 48, 49, 50].

Education and training: training coaches, referees, and medical personnel on the importance of CPR and the timely use of AEDs is critical. Research should explore the most effective strategies for implementing educational programs that increase awareness and preparedness for CC [51, 52].

Screening and identification of at-risk individuals: although CC is often unpredictable, research could focus on identifying potential risk factors through advanced cardiovascular screening, including genetic testing and electrophysiological assessments, to identify potentially susceptible individuals [53, 54].

Data collection and national registries: the creation and analysis of national registries dedicated to CC can provide valuable data to better understand the incidence, risk factors and effectiveness of preventive measures. These registries can also help monitor the impact of new guidelines and emerging technologies.

Technological innovations: the integration of wearable technologies and advanced sensors could offer new opportunities to monitor athletes’ physiological parameters in real time and detect early warning signals, allowing for timely interventions.

Interdisciplinary collaborations: promoting collaborations between cardiologists, engineers, educators and sports organizations is essential to develop integrated and multidisciplinary approaches in the prevention and management of CC.

Future research on commotio cordis should take a holistic approach, combining technological innovations, education, screening, and interdisciplinary collaboration to reduce the incidence and improve outcomes of this potentially fatal event.

## 3. Conclusions 

CC is a rare but serious cause of SCDY, often in sports settings but not exclusively. Its unpredictable nature and the absence of structural cardiac abnormalities make timely recognition and immediate intervention with CPR and defibrillator essential. Furthermore, since genetic evaluations are not routinely performed in autopsy practice, determining the exact number of CC cases is extremely difficult.

Despite progress in terms of awareness and prevention, critical issues remain, especially in non-sporting contexts. It is essential to promote widespread access to AEDs, resuscitation training and the development of more effective protective devices. An integrated approach between research, education and field preparation is crucial to reduce the incidence and improve the outcomes of this dramatic condition.
